# Effectiveness of primary care psychological therapy services for the treatment of depression and anxiety in people living with dementia: Evidence from national healthcare records in England

**DOI:** 10.1016/j.eclinm.2022.101692

**Published:** 2022-10-14

**Authors:** Georgia Bell, Celine El Baou, Rob Saunders, Joshua E. J Buckman, Georgina Charlesworth, Marcus Richards, Barbara Brown, Shirley Nurock, Stuart Michael, Paul Ware, Elisa Aguirre, Miguel Rio, Claudia Cooper, Stephen Pilling, Amber John, Joshua Stott

**Affiliations:** a Adapt Lab, Research Department of Clinical, Educational and Health Psychology, UCL, London, UK; b Centre for Outcomes Research and Effectiveness, Research Department of Clinical, Educational and Health Psychology, UCL, London, UK; c iCope – Camden & Islington NHS Foundation Trust, St Pancras Hospital, London, UK; d MRC Unit for Lifelong Health and Ageing at UCL, UCL, London, UK; e North East London NHS Foundation Trust (NELFT), London, UK; f Department of Electronic and Electrical Engineering, UCL, London, UK; g Centre for Psychiatry and Mental Health, Wolfson Institute for Population Health, Queen Mary University of London, London, UK; h East London NHS Foundation Trust, London, UK; i Camden & Islington NHS Foundation Trust, St Pancras Hospital, London, UK

**Keywords:** Dementia, Depression, Anxiety, National healthcare data

## Abstract

**Background:**

Depression and anxiety are common and deleterious in people living with dementia (PLWD). It is currently unknown whether routinely provided psychological therapy can help reduce these symptoms in PLWD. This study aimed to investigate improvements in depression and anxiety symptoms over the course of therapy offered in primary care psychological therapy services in PLWD and to compare outcomes to people without dementia.

**Methods:**

National data from Improving Access to Psychological Therapies services (IAPT) across England linked with Hospital Episode Statistics data, the Mental Health Services Dataset, and HES-ONS mortality data were used to identify 1,549 PLWD who completed a course of psychological treatment in IAPT between 2012-2019 and a propensity score matched control group without identified dementia. Outcome measures included pre-post intervention changes in depression (PHQ-9) and anxiety (GAD-7) symptoms and therapy outcomes (reliable improvement, recovery, deterioration).

**Findings:**

Symptoms of depression (*t*(1548)=31·05, *p*<·001) and anxiety (*t*(1548)=30·31, *p*<·001) improved in PLWD over the course of psychological therapy with large effect sizes (depression: *d*=-0·83; anxiety: *d*=-0·80). However, PLWD were less likely to reliably improve (OR=·75, 95%CI[·63,·88], p<·001) or recover (OR=·75, 95%CI[·64,·88], *p*=·001), and more likely to deteriorate (OR=1·35, 95%CI[1·03,1·78], *p=*·029) than a matched control sample without dementia.

**Interpretation:**

Psychological therapy may be beneficial for PLWD with depression or anxiety, but it is currently not as effective as for people without dementia. More research is needed to improve access to psychological therapies and to understand this discrepancy and how therapies can be adapted to further improve outcomes.

**Funding:**

This work was supported by the Alzheimer's Society.

**Research in context**

**Evidence before this study**

Efficacy of psychological therapies in reducing symptoms of depression and anxiety in people living with dementia (PLWD) has been examined in randomised control trials (RCTs), however the effectiveness of primary care psychological therapy services in improving anxiety and depression in PLWD has yet to be examined in a naturalistic setting. A total of 159 papers were identified from a literature search in Pubmed (from inception to 28/06/22) using the following title/abstract search terms: (anxi* OR depress*) AND (therap* OR interven*) AND (demen* OR Alzheimer*) AND ((primar* NEAR care*) OR routine* OR (service* NEAR (mental OR psycholog*))). No relevant studies were identified.

**Added value of this study**

This is the first study to investigate psychological therapy outcomes for a large sample of PLWD using routinely collected national data. Our results support the treatment of anxiety and depression in PLWD within primary care psychological therapy services. This is critical given the high rates of anxiety and depression in PLWD and the adverse health, social, and economic cost of these comorbidities as well as the lack of strong evidence for the efficacy of other treatment options such as antidepressants in PLWD.

**Implications of all the available evidence**

Our work combined with positive findings from previous RCTs suggest that primary care psychological therapy services can be useful for reducing symptoms of depression and anxiety in PLWD. However, more research is needed to understand how outcomes can be improved. This research has important implications for encouraging more referrals and adaptations to standard care to increase access and improve psychological therapy outcomes for PLWD with depression or anxiety.

Alt-text: Unlabelled box

## Introduction

Depression, anxiety, and dementia are major contributors to global health-related burden individually, making them key issues for public health.^1^^,^^2^ Globally, anxiety and depression are estimated to affect 3·6% and 4·4% of the population respectively and mental health problems are estimated to cost the global economy $2·5 trillion each year.^3^^,^^4^ Depression and anxiety are more common in people living with dementia (PLWD), with prevalence estimates of 38% for both depression and anxiety in mild dementia.^5^ Furthermore, apart from the subjective distress experience arising from these comorbid problems, anxiety and depression in PLWD are associated with numerous adverse outcomes, such as lower quality of life, faster cognitive decline, earlier institutionalisation, and greater carer distress.^6^, ^7^, ^8^, ^9^, ^10^

Psychological therapies offered in primary care mental health services are a recommended first line treatment for depression and anxiety (e.g., recommended by NICE in the UK), including for PLWD.^11^, ^12^, ^13^ Patients have reported an approximate 3-to-1 preference for psychological therapy over pharmacological interventions, and in PLWD specifically, non-pharmacological interventions appear to be more effective in reducing depressive symptoms than antidepressant medication.^14^^,^^15^ Previous reviews have generally found evidence from randomised controlled trials (RCTs) for the efficacy of psychological therapies for reducing symptoms of depression, anxiety, and psychological distress in PLWD.^16^, ^17^, ^18^ However, given that there may be systematic differences between PLWD who take part in research studies and those who receive clinical care, it is unclear how representative these outcomes are for PLWD in routine clinical practice.^19^ Understanding this is critical for informing service design, development, and implementation. However, to our knowledge, no previous study has examined outcomes for PLWD in routinely delivered psychological therapy services anywhere in the world. Consequently, in line with MRC guidance for evaluating complex interventions, this study uses a naturalistic design to examine psychological therapy outcomes across all services in a nationally provided primary care psychological therapy programme (IAPT).^20^^,^^21^ Improving Access to Psychological Therapies (IAPT) services are freely available across all of England via the NHS, and all offer a variety of evidence-based psychological therapies for common mental health problems delivered by trained professionals, with recent policy guidance requiring services to accept PLWD.^12^^,^^21^^,^^22^

This study aims to:

1 Examine the effectiveness of routinely delivered psychological therapies in IAPT for reducing symptoms of depression or anxiety in PLWD
2 Investigate whether the degree of improvement in therapy outcomes in PLWD differs to people without identified dementia

## Methods

### Data

The MODIFY study utilises data from patients seen for psychological therapy in every IAPT service across all 211 clinical commissioning group areas in England between 2012 to 2019 linked with Hospital Episode Statistics (HES) data, the Mental Health Services Dataset (MHSDS), and HES-ONS mortality data.^21^^,^^23^, ^24^, ^25^ These data were linked using a key provided by NHS Digital. This dataset includes information on demographic (e.g., gender, age, ethnicity), psychological therapy (e.g., referral and assessment dates, treatment outcomes) and other healthcare (e.g., inpatient and outpatient records, diagnosis and treatment, cause and place of death) variables for individual patients across England. See Supplementary A for more information. Ethical approval was not required for this study. The databases did not collect consent. The data were pseudonymised and the data were legally processed under GDPR article 61(e) (relating to public interest) and 9(2)j (relating to scientific research). This legal basis for processing the data was approved by NHS Digital's Independent Group Advising on the Release of Data (IGARD) committee.

### Participants

Participants included people who completed a course of psychological therapy in IAPT between 2012 and 2019 and have a record in IAPT and a linked record in HES/MHSDS to identify dementia. A standard set of exclusion criteria used in studies of IAPT samples were applied in order to identify a sample that received psychological therapy for depression or an anxiety disorder: 1) did not have a course of treatment (defined as two or more sessions of psychological therapy), 2) did not meet the clinical cut-off for ‘caseness’ for depression (10+ on PHQ-9) or generalised anxiety disorder (8+ on GAD-7), 3) had a primary diagnosis for which there is no evidence-based psychological therapy offered in IAPT (e.g., severe mental illnesses such as schizophrenia and bipolar disorder, alcohol dependency, bereavement), 4) were still in treatment, 5) were missing data for baseline or follow-up measures on the Patient Health Questionnaire 9-item (PHQ-9) or Generalized Anxiety Disorder Scale 7-item (GAD-7) (as pre-post data on these measures are completed for approximately 99% of IAPT patients, it was expected that this would result in very few participants being excluded (*n =* 3133)).^21^^,^^26^^,^^27^ We also excluded participants who received a dementia diagnosis during or after IAPT treatment. Where participants had more than one episode of treatment in an IAPT service during the time period of data collection relevant to this study, only data for the first course of treatment were used. Out of a total 2,515,402 patients who received treatment in IAPT between 2012 to 2019, 1,945,323 patients were eligible and included in analyses, of whom 1549 (0·08%) had a diagnosis of dementia prior to attending IAPT.

### Measures

#### Demographic and therapy measures

Self-reported demographic information was available from routinely collected IAPT data, including gender, age at referral, ethnicity (consistent with ONS codes), index of multiple deprivation (IMD) decile (1 represents the most deprived 10% of geographical areas in England and 10 represents the least deprived 10%), and employment status (employed vs unemployed).^28^ Psychological therapy and health measures were available from IAPT data. These included number of therapy sessions attended, year of first and last therapy sessions, and self-reported measures of whether service users were taking psychotropic medication and whether they had a long-term health condition (LTC). Additionally, waiting times from referral to assessment and assessment to treatment were calculated from appointment dates.

#### Clinical measures

Depression and anxiety measures were taken from the IAPT dataset.^29^ Depression was assessed using the Patient Health Questionnaire 9-item (PHQ-9), with a ‘caseness’ threshold score of ≥10.^30^ Caseness refers to a level of symptoms likely to be sufficient for to meet diagnostic criteria for the measured disorder. Generalised anxiety was assessed using the Generalized Anxiety Disorder Scale 7-item (GAD-7), with a ‘caseness’ threshold score of ≥8.^31^ For patients with a diagnosis of a specific anxiety disorder (e.g., Social Phobia or Panic Disorder), ‘anxiety disorder specific measures’ (ASDMs) were used (Supplementary B). All-cause dementia status was identified using ICD-10 dementia codes from HES and MHSDS data.^32^

#### Outcome measures

Primary outcomes were based on nationally determined outcome metrics used in IAPT^29^:

• *Reliable improvement*: a reduction in depression or anxiety symptoms from the first to last attended treatment session that exceeds the threshold for error of measurement on the corresponding symptom scale (≥6 points on PHQ-9, ≥4 points on GAD-7; see Supplementary B for ADSM cut-offs)
• *Reliable recovery*: reliable improvement and ending treatment below the threshold for ‘caseness’ on both the measure of depression and anxiety (as described above)
• *Reliable deterioration*: an increase in depression or anxiety symptoms from the first to last attended treatment session by at least the magnitude of the threshold for the error of measurement (see reliable improvement above)

Secondary outcomes included pre-post change on measures of depression (PHQ-9), generalised anxiety (GAD-7), and functional impairment (Work and Social Adjustment Scale; WSAS).^33^

### Statistical analysis

Analyses were conducted using STATA 17.^34^ First, comparisons of baseline characteristics between the dementia and control group were conducted using independent t-tests and chi-square tests. Missing data for categorical variables were dummy coded to retain a larger sample. Due to a small number of extreme values, waiting time variables were winsorized at the top 99% to reduce the influence of outliers. Additionally, t-tests and chi-square tests were also conducted to compare outcome measures between groups. To understand the representativeness of PLWD accessing IAPT, we first calculated the percentage of people with dementia in the MODIFY sample across a) all age groups and b) aged 65+. To contextualise this, we conducted an analysis to approximate the representation using national dementia prevalence figures for mild-moderate dementia in older adults, the prevalence of depression and anxiety in mild-moderate dementia, and the prevalence of depression in a general older population (Supplementary C).^5^^,^^35^^,^^36^ Next, paired t-tests were used to investigate pre-post differences in PHQ-9 and GAD-7 scores for the dementia group. Given the lack of a control group of PLWD not receiving IAPT therapy in the dataset, we identified comparison groups from a recent systematic review that investigated the effectiveness of psychosocial interventions for comorbid depression or anxiety in PLWD using evidence from RCTs.^16^ Studies were selected where appropriate data were available for pre- and post-intervention measures of either anxiety or depression for the control group. These findings were used to contextualise the current findings by calculating standardised mean differences.

Logistic regression models were fitted to explore associations between dementia and primary outcomes, and linear regression models were used to explore secondary outcomes. These models were first run using the full sample, then again using a propensity score (PS) matched sample. PLWD were matched with control participants without identified dementia on all variables listed in Table 1 (except number of sessions) using *psmatch2* (see Supplementary D for PS matching model)*.*^37^ The caliper was set at 0·001, in line with previous research using PS matching with IAPT data.^26^ Where a control was identified as an appropriate match for more than one participant in the dementia sample, matching with replacement was applied. These were weighted and used in the analysis, no control was matched to more than 2 people from the dementia sample (maximum weight = 2). Sensitivity analyses excluding people who were diagnosed with dementia before the age of 65 were also conducted. Additionally, subgroup analyses were performed to explore associations between dementia and outcomes across treatment intensity: Low intensity only (e.g., guided self-help, computerised cognitive behavioural therapy), High intensity only (e.g., CBT, interpersonal psychotherapy), and Mixed intensity (patients who were either stepped up or stepped down during their episode of treatment).^21^ Finally, as outcomes may differ between IAPT services, multilevel logistic regression (primary outcomes) and multilevel mixed-effects linear regression (secondary outcomes) models with random intercepts were used to explore clustering effects by Clinical Commissioning Group (see Supplementary E for categories).

**Table 1:** Comparison of baseline characteristics.

		Before PS matching			After PS matching		
		Dementia group (*n =* 1549)	Control (*n =* 1,943,774)		Dementia group (*n =* 1351)	Control (*n =* 1329)	
		N (%)	N (%)	*p*-value	N (%)	N (%)	*p*-value
Gender	Male	656 (42·35%)	638,827 (32·87%)	<·001	577 (42·71%)	548 (41·23%)	·74
	Female	889 (57·39%)	1,298,144 (66·78%)		771 (57·07%)	778 (58·54%)	
	Missing / preferred not to answer	4 (0·03%)	6,803 (0·35%)		3 (0·22%)	3 (0·23%)	
Ethnicity (ONS)	White	1,231 (79·47%)	1,592,990 (81·95%)	<·001	1,082 (80·09%)	1,070 (80·51%)	·98
	Mixed	16 (1·03%)	37,581 (1·93%)		15 (1·11%)	15 (1·13%)	
	Asian	69 (4·45%)	82,892 (4·26%)		56 (4·15%)	52 (3·91%)	
	Black	55 (3·55%)	48,121 (2·48%)		37 (2·74%)	42 (3·16%)	
	Other	22 (1·42%)	20,958 (1·08%)		20 (1·48%)	17 (1·28%)	
	Missing / preferred not to answer	156 (10·07%)	161,232 (8·29%)		141 (10·44%)	133 (10·01%)	
Employment status	Employed	1,016 (65·59%)	1,423,631 (73·24%)	<·001	897 (66·40%)	905 (68·10%)	·12
	Unemployed	397 (25·63%)	404,671 (20·82%)		362 (26·79%)	316 (23·78%)	
	Missing / preferred not to answer	136 (8·78%)	115,472 (5·94%)		92 (6·81%)	108 (8·13%)	
LTC Case	No	393 (25·37%)	1,087,647 (55·96%)	<·001	353 (26·13%)	363 (27·31%)	·54
	Yes	771 (49·77%)	446,768 (22·98%)		692 (51·22%)	687 (51·69%)	
	Missing	385 (24·85%)	409,359 (21·06%)		306 (22·65%)	279 (20·99%)	
Psychotropic medication	Prescribed – not taking	39 (2·52%)	92,403 (4·75%)	<·001	38 (2·81%)	35 (2·63%)	·52
	Prescribed and taking	826 (53·32%)	916,808 (47·17%)		754 (55·81%)	734 (55·23%)	
	Not prescribed	427 (27·57%)	755,864 (38·89%)		383 (28·35%)	361 (27·16%)	
	Missing / preferred not to answer	257 (16·59%)	178,699 (9·19%)		176 (13·03%)	199 (14·97%)	
Index of Multiple Deprivation (IMD) Decile	1	231 (14·91%)	208,662 (10·73%)	<·001	196 (14·51%)	202 (15·20%)	·89
	2	183 (11·81%)	209,808 (10·79%)		148 (10·95%)	150 (11·29%)	
	3	184 (11·88%)	208,714 (10·74%)		155 (11·47%)	153 (11·51%)	
	4	154 (9·94%)	205,407 (10·57%)		123 (9·10%)	125 (9·41%)	
	5	153 (9·88%)	193,805 (9·97%)		139 (10·29%)	132 (9·93%)	
	6	152 (9·81%)	185,660 (9·55%)		139 (10·29%)	137 (10·31%)	
	7	124 (8·01%)	177,192 (9·12%)		112 (8·29%)	114 (8·58%)	
	8	120 (7·75%)	171,647 (8·83%)		114 (8·44%)	126 (9·48%)	
	9	104 (6·71%)	165,031 (8·49%)		95 (7·03%)	79 (5·94%)	
	10	95 (6·13%)	153,640 (7·90%)		91 (6·74%)	85 (6·40%)	
	Missing	49 (3·16%)	64,208 (3·30%)		39 (2·89%)	26 (1·96%)	
Year of first appointment	2012	19 (1·23%)	60,387 (3·10%)	<·001	15 (1·11%)	12 (0·90%)	·99
	2013	75 (4·84%)	218,483 (11·24%)		66 (4·89%)	64 (4·82%)	
	2014	163 (10·52%)	290,669 (14·95%)		144 (10·66%)	140 (10·53%)	
	2015	278 (17·95%)	336,423 (17·31%)		227 (16·80%)	226 (17·01%)	
	2016	315 (20·34%)	338,177 (17·40%)		273 (20·21%)	263 (19·79%)	
	2017	310 (20·01%)	319,400 (16·43%)		274 (20·28%)	286 (21·52%)	
	2018	317 (20·46%)	314,087 (16·16%)		284 (21·02%)	272 (20·47%)	
	2019	72 (4·65%)	66,148 (3·40%)		68 (5·03%)	66 (4·97%)	

		Mean (SD)	Mean (SD)	*p*-value	Mean (SD)	Mean (SD)	*p*-value
Age at referral		65·92 (16·19)	40·31 (14·71)	<·001	65·33 (15·76)	65·45 (15·21)	·84
Baseline PHQ-9		15·56 (5·78)	15·72 (5·62)	·26	15·74 (5·80)	15·71 (5·85)	·90
Baseline GAD-7		12·97 (5·11)	14·28 (4·45)	<·001	13·13 (5·07)	13·26 (4·94)	·52
Waiting time: referral to assessment (weeks)		3·26 (4·20)	3·24 (4·28)	·83	3·39 (4·30)	3·29 (4·44)	·54
Waiting time: assessment to treatment (weeks)		6·89 (7·35)	6·68 (7·11)	·26	6·98 (7·37)	6·89 (7·33)	·76
Number of sessions^a^		5·53 (3·98)	6·51 (4·577)	<·001	5·87 (4·00)	6·43 (4·30)	<·001

Independent t-tests were used for continuous variables and chi-square tests were used for categorical variables.

a Note, number of sessions was not included in the PS matching algorithm.

### Role of the funding source

The funders of the study were not involved in the study design, data analysis, interpretation of data, writing of the report, and in the decision to submit the paper for publication. All authors had full access to all data in the study and accept the responsibility to submit for publication.

## Results

### Sample characteristics

Comparisons of participants with complete data and missing data on key variables for both dementia and control group are presented in Supplementary F. Sample characteristics (demographic and therapy variables) for PLWD and the control group without identified dementia are presented in Table 1. Prior to matching, PLWD were older at age of referral and had lower baseline scores on GAD-7 but not on the PHQ-9. There were also differences between groups for gender, ethnicity, employment status, LTC case, psychotropic medication, IMD decile, and appointment year. No differences in waiting times (referral to assessment, assessment to treatment) between groups were identified. After propensity score (PS) matching, there were no significant differences in baseline characteristics between groups. Comparisons of outcome measures (primary and secondary) for PLWD and the control group are presented in Table 2.

**Table 2:** Comparison of outcomes before and after PS matching.

Before PS matching					
	Dementia		Control		
Primary outcomes	Total N	N (%)	Total N	N (%)	p
**Reliable improvement**	1,544	951 (61·59%)	1,936,805	1,364,952 (70·47%)	<·001
**Reliable recovery**	1,375	536 (38·98%)	1,690,479	756,604 (44·76%)	<·001
**Reliable deterioration**	1,543	153 (9·92%)	1,927,859	124,240 (6·44%)	<·001
**Secondary outcomes**	**Total N**	**M (SD)**	**Total N**	**M (SD)**	**p**
**PHQ-9 Change**	1,549	5·24 (6·64)	1,943,774	6·35 (6·56)	<·001
**GAD-7 Change**	1,549	4·39 (5·70)	1,943,774	5·85 (5·91)	<·001
**WSAS Change**	1,113	3·90 (9·63)	1,296,047	6·00 (9·60)	<·001
**After PS matching**					
	**Dementia**		**Control**		
**Primary outcomes**	**Total N**	**N (%)**	**Total N**	**N (%)**	**p**
**Reliable improvement**	1,348	853 (63·28%)	1,326	923 (69·61%)	·001
**Reliable recovery**	1,197	482 (40·27%)	1,169	550 (47·05%)	·001
**Deterioration**	1,347	131 (9·73%)	1,322	98 (7·41%)	·033
**Secondary outcomes**	**Total N**	**M (SD)**	**Total N**	**M (SD)**	**p**
**PHQ-9 Change**	1,351	5·48 (6·73)	1,329	6·60 (6·64)	<·001
**GAD-7 Change**	1,351	4·58 (5·79)	1,329	5·48 (6·07)	<·001
**WSAS Change**	993	4·27 (9·74)	988	5·70 (9·40)	<·001

Independent t-tests were used for continuous outcomes and chi-square tests were used for categorical outcomes.

### Improvement in therapy outcomes in PLWD

Pre and post intervention symptoms of depression (pre: *M* = 15·56, *SD* = 5·78; post: *M* = 10·32, *SD* = 6·83) and generalised anxiety (pre: *M* = 12·97, *SD* = 5·11; post: *M* = 8·58, *SD* = 5·87) in PLWD improved on average over the course of IAPT therapy (depression: *t*(1548) = 31·05, *p* < ·001; anxiety: *t*(1548) = 30·31, *p* < ·001). This constituted a large effect size for decreases in symptoms of depression (*d* = -0·83) and anxiety (*d* = -0·80). For context, findings from RCTs examining non-pharmacological interventions for anxiety and depression in PLWD with clinical depression or anxiety are presented in Supplementary G.^16^ Two comparison studies were identified for anxiety measures (Intervention *d* = -0·13 and -1·42; Control *d* = -0·26 and -0·08) and five comparison studies were identified for depression with effect sizes ranging from -0·24 to -0·60 for intervention and 0·04 to -0·40 for control groups.

### Differences in outcomes between PLWD and matched control group without dementia

Of the 1,368 PLWD with complete data available for all continuous variables used in the matching algorithm, 17 were unable to be matched. The final matched sample consisted of 1351 PLWD and 1329 matched controls without identified dementia. Primary and secondary outcomes are presented in Table 3. For primary outcomes, there was evidence that PLWD had lower likelihood of reliable improvement (OR = ·75, 95% CI [·63, ·88], *p* < ·001) and reliable recovery (OR = ·75, 95% CI [·64, ·88], *p =*·001) and higher likelihood of reliable deterioration (OR = 1·35, 95% CI [1·03, 1·78], *p =*·029) in symptoms compared to a PS matched control sample without identified dementia. Results remained consistent when controlling for all matched variables and number of sessions attended, with the exception of deterioration (OR = 1·31, 95% CI [·99, 1·75], *p =* 062). For secondary outcomes, having dementia was associated with less change in depression (b = -1·14, se = ·26, *p <* ·001), generalised anxiety (b = -·92, se = ·23, *p <* ·001), and general functioning (WSAS) (b = -1·38, se = ·43, *p =* ·001), than having no identified dementia. Findings from multilevel models for primary and secondary outcomes were very similar to single-level models, with intraclass correlations coefficients indicative of differences between service regions accounting for less than 1% of the variation in both primary and secondary outcomes (Supplementary H).

**Table 3:** Primary and secondary outcomes.

Primary outcomes	Reliable improvement				Reliable recovery				Reliable Deterioration			
	N	OR	95% CI	p	N	OR	95% CI	p	N	OR	95% CI	p
**Full sample (unadjusted)**	1,938,349	·67	·61, ·74	<·001	1,691,854	·79	·71, ·88	<·001	1,929,402	1·60	1·35, 1·89	<·001
**PS matched (unadjusted)**	2696	·75	·63, ·88	<·001	2383	·75	·64, ·88	·001	2691	1·35	1·03, 1·78	·029
**PS matched (adjusted)**^a^	2696	·78	·66, ·93	·004	2378	·79	·66, ·94	·006	2685	1·31	·99, 1·75	·062
**Secondary outcomes**	**PHQ-9 Change**				**GAD-7 Change**				**WSAS Change**			
	N	b	se	p	N	b	se	p	N	b	se	p
**Full sample (unadjusted)**	1,945,323	-1·11	·17	<·001	1,945,323	-1·46	·15	<·001	1,297,160	-2·10	·29	<·001
**PS matched (unadjusted)**	2702	-1·14	·26	<·001	2702	-·92	·23	<·001	2000	-1·38	·43	·001
**PS matched (adjusted)**^a^	2702	-·93	·23	<·001	2702	-·65	·20	·001	2000	-1·34	·42	·002

Logistic regression models were used for primary outcomes and linear regression models were used for secondary outcomes.

a Adjusted for all matched variables (gender, ethnicity, employment status, LTC case, psychotropic medication, IMD decile, year of first appointment, age at referral, baseline PHQ-9, baseline GAD-7, waiting times referral to assessment, waiting time assessment to treatment) and number of IAPT sessions attended.

### Age of dementia diagnosis sensitivity analyses

People diagnosed with dementia before the age of 65 (PLWD<65) accounted for 44·16% of the dementia sample. There were differences in reliable improvement (64% vs 58%) and reliable recovery (46% vs 31%) outcomes between PLWD diagnosed with dementia aged 65+ and PLWD<65, but not for reliable deterioration or secondary outcomes (Supplementary I). Sensitivity analyses exploring differences in outcomes between PWLD (diagnosed <65 only/65+ only) and matched controls without dementia were in line with main models for both groups (Supplementary J and K), except for reliable deterioration and WSAS change.

### Treatment intensity subgroup analyses

The PS matching algorithm was rerun on each subsample (High only, Low only, Mixed). Results are presented in Supplementary L. No significant results were found in the mixed intensity group. Findings for the high only and low only groups were largely in line with main models, with the exception of reliable deterioration which was non-significant in the high intensity only group and WSAS change which was non-significant in the low intensity only group.

## Discussion

For those accessing primary care psychological therapy, depression and anxiety scores in PLWD significantly change over the course of IAPT therapy with large effect sizes. However, PLWD are less likely to reliably improve or reliably recover than people without dementia. While outcomes are worse in PLWD than a matched sample without dementia, the difference pre- and post-intervention in PLWD does appear to be clinically meaningful.^38^ While causality cannot be established by this study design, our work does provide initial evidence that psychological therapies offered in primary care mental health services may be effective in reducing symptoms of depression and anxiety in PLWD. Moreover, it appears that around 62% of PLWD reliably improve and 40% reliably recover following IAPT therapy.

The present findings should be interpreted in the context of a somewhat selective sample as we estimate that PLWD may be underrepresented in IAPT by 1·5-9 fold (Supplementary C) and our sample had an overrepresentation of PLWD diagnosed before age 65 (young-onset accounts for ∼9% of dementia cases).^39^ However, the large effect sizes found were in line with and in many cases larger than findings from RCTs which arguably include a more selective samples and subdividing the results by older and younger onset groups did not drastically alter findings. Further, given previous findings that older adults are more likely to improve and recover following therapy than working-age adults, it is also notable that prior to PS matching the control sample had a mean age 25 years younger than the dementia sample, yet results remained consistent pre-post matching.^26^ While it is useful to know about the representation of PLWD in IAPT, these findings are the first to demonstrate the important principle that PLWD can benefit from primary care psychological therapies. We do expect though that effect sizes may be attenuated if the sample were more representative of PLWD as a whole.

Given the poorer outcomes for PLWD when compared to those without dementia, adaptations may be required to make outcomes more comparable.^17^ As data were derived from natural therapy settings, it is possible that some adaptations were made for PLWD during their treatment, although data regarding this were unavailable. Key adaptations might be to adjust therapy structure to accommodate cognitive difficulties or involvement of carers in the therapy process, both of which some IAPT therapists already do.^40^ Additionally, as cognitive and behavioural symptoms differ between different types of dementia and in younger and older people (with non-memory led dementia more common in young-onset), it is important that specific adaptations are tailored to the individual.^41^

To our knowledge, this is the largest study to examine psychological therapy outcomes for PLWD. Further, this is the first study to investigate and support the utility of current UK recommendations that such therapies be offered to PLWD in a primary care context.^12^^,^^22^ Limitations include the inability to infer causal relationships between receipt of therapy and symptom improvement and outcome measurement issues as neither the PHQ9 nor GAD7 have been validated for use in a pure sample of PLWD. However, the PHQ9 has been shown to have good validity in a sample with a high proportion of PLWD and in our sample responses to self-report questionnaires were routinely checked and reviewed with patients by clinicians trained in diagnosis, outcome measurement, and the therapy they were conducting.^42^^,^^43^ Next, due to the stepped-care model used in IAPT with patients often receiving a range of evidence-based treatments within an episode of care, we could not reliably investigate type of psychological therapy. Moreover, whilst CBT-related therapies can be offered to all patients in IAPT, other types of therapies are only offered to people with specific mental health diagnoses (e.g., interpersonal psychotherapy or psychodynamic psychotherapy for people with depression, EMDR for people with PTSD).^21^ Instead, we investigated associations between dementia and therapy outcomes across treatment intensity as this is a more important distinction in this setting and more inclusive across mental health diagnoses. Another limitation may be that the identification of dementia in this study was based on linked records, thus some PLWD attending IAPT (those who did not have a linked record) may have been missed. If this did bias findings, it is likely that the proportion of PLWD with a linked IAPT/HES record is higher than in people without dementia; our study might therefore reflect a bias towards a higher level of representation of PLWD than is actually the case. Further, using HES data required relying on formal dementia diagnosis. As such, we could not capture suspected but as yet undiagnosed dementia or cases where dementia was not recorded in HES, although previous research suggests HES recording is valid.^44^ Finally, we could not account for dementia severity at the time of psychological treatment. It is likely that the PLWD treated in IAPT were presenting with mild symptoms of dementia, however data were not available to explore this.

Our work has important implications for public health. The utility of our findings are important for encouraging referrals of PLWD into primary care psychological therapy services, as our work suggests that these services are likely to be useful in treating anxiety and depression in PLWD. Additionally, in light of PLWD's under-representation, improving access to these services is essential. This could perhaps be based on understanding barriers and facilitators identified in previous work^40^; however, further work is needed to understand and optimise pathways into (e.g., referral, waiting times) and through (e.g., number of sessions, treatment type) therapy for PLWD. This research also highlights the need to identify appropriate adaptations that could improve therapy outcomes for PLWD and ensure that clinicians have adequate training for working with PLWD and implementing these adaptations. Currently, dementia-specific training is not routinely offered to clinicians in IAPT, with clinicians often feeling unsupported in working with PLWD.^40^ Finally, we acknowledge that there is a lot of variation in dementia, thus psychological therapies may not suit everyone. As such, appropriateness for services should be determined prior to referral and a range of interventions should be available to suit different preferences and levels of cognitive impairment. More research is needed to explore which factors (e.g., sociodemographic, dementia type, therapy variables) are associated with better therapy outcomes in PLWD to facilitate this training and allow understanding of who will benefit.

Psychological therapies offered in primary care mental health services may be beneficial for reducing symptoms of depression and anxiety in PLWD; however, PLWD appear less likely to experience improvement in symptoms or recover from depression or anxiety, and more likely to experience symptomatic deterioration compared to those without dementia. Given current public health recommendations, research exploring therapy outcomes in PLWD using data from naturalistic settings is crucial for understanding whether these services are effective. Greater insight into why there is a difference in therapy outcomes between PLWD and people without dementia could help inform adaptations in services to improve these outcomes for PLWD.

## Contributors

All authors were involved in the conceptualisation and design of the study. GB, CEB, AJ, JS, RS, and JB contributed to the methodology and formal analysis. GB, AJ, and JS assessed and verified the underlying data reported in the manuscript. All authors contributed to the manuscript writing, reviewing, and approved the final version. All authors had full access to all data in the study and accept the responsibility to submit for publication.

## Data sharing statement

All data used for this study are available upon successful application to NHS Digital via the Data Access Request Service (DARS): https://digital.nhs.uk/services/data-access-request-service-dars. Data fields can be accessed via NHS Digital data dictionary: https://www.datadictionary.nhs.uk/.

## Declaration of interests

GB, JS, and AJ are supported by the Alzheimer's Society (AS-PG-18-013). JB is supported by the Royal College of Psychiatrists. CEB has been a consultant to Eli Lilly and Company. RS held unrelated honorary position with NHS England, time was compensated through financial support to employing institution. JS has been a consultant to NHS Wales Shared Services Partnership and is involved in unrelated research projects funded by NIHR Public Health Research, Dunhill Medical Trust, and ESRC/NIHR.

## Appendix Supplementary materials

Image, application 1
