# Supplementary material for: Effectiveness of primary care psychological therapy services for the treatment of depression and anxiety in people living with dementia: Evidence from national healthcare records in England
Source: eClinicalMedicine. 2022 Oct 14;52:101692. doi: 10.1016/j.eclinm.2022.101692 (PMC9596302; doi:10.1016/j.eclinm.2022.101692)
Supplement: Supplementary file 1 [file mmc1.pdf]

Supplementary Materials

Table of contents

A) MODIFY Study dataset 2

B) Thresholds for Anxiety disorder specific disorders (ADSM) 3

C) Proportion of PLWD in IAPT 4

D) Propensity score matching model 5

E) Clinical commissioning group categories 6

F) Comparison of participants with complete and missing data 8

G) Contextualising change in therapy outcomes in PLWD with previously published studies 9

H) Multilevel models 10

I) Comparison of outcomes between age of dementia diagnosis groups (65+ vs >65 years) 11

J) Sensitivity analyses: Primary and secondary outcomes for PLWD<65 and matched controls 12

K) Sensitivity analyses: Primary and secondary outcomes for PLWD65+ and matched controls 13

L) Sensitivity analyses: Treatment intensity 14

References 15

A) MODIFY Study dataset

**IAPT dataset (1)**	Routinely collected data for every patient seen in IAPT services across all 211 clinical commissioning group areas in England between 2012 to 2019. Includes demographic (e.g., gender, age, ethnicity), therapy (e.g., referral and assessment dates, treatment information at each appointment) and outcome (improvement, recovery, deterioration) information for individual patients.
**Hospital Episode Statistics dataset (2)**	Admitted Patient Car and Outpatient datasets from all National Health Service (NHS) hospitals across England. Includes demographic (e.g., age, ethnicity, gender), geographical (e.g., residential area, area treatment received), administrative (e.g., dates of admission and discharge), and clinical (e.g., diagnoses, treatments, operations) information for individual patients.
**Mental Health Services dataset (3)**	Previously known as the Mental Health Minimum Dataset (MHMDS) and the Mental Health and Learning Disability Dataset (MHLDDS). Includes data from secondary care services (e.g., provided in hospitals, outpatient clinics, in the community) for mental illness, learning disability, autism, and other neurodevelopmental conditions.
**HES-ONS Mortality dataset (4)**	Linked information from HES and Office of National Statistics (ONS) mortality data. Includes cause, date, and place of death (both in and out of hospital).

B) Thresholds for Anxiety disorder specific disorders (ADSM)

**ADSM (5)**	**Caseness**	**Change (improvement/deterioration)**
Agoraphobia Morbidity Inventory	>2·3	0·73
Social Phobia Inventory	19	10
Panic Disorder Severity Scale	8	>5
Impact of Events Scale (PTSD)	33	9
Obsessive Compulsive Inventory	40	32
Health Anxiety Inventory	18	4

C) Proportion of PLWD in IAPT

In our study, PLWD made up 0·08% of the full sample and 0·74% of participants aged 65 and over. To calculate representativeness, we compared the difference in the observed proportion of PLWD aged 65+ who had treatment in IAPT and the expected proportion of PLWD aged 65+ who may be eligible for such therapy. The lower bound estimate of expected access (1·14%) was calculated using national dementia prevalence estimates for mild to moderate dementia (3%) (6) and the prevalence estimates of depression and anxiety in mild to moderate dementia (38%) (7). Given the higher level of anxiety and depression in PLWD, this is likely to be an underestimate. To calculate the upper bound estimate of expected access (6·69%), we also accounted for depression prevalence in a general older population (17%) (8). This may suggest that the number of PLWD aged 65+ in IAPT is between 1·5 and 9 times lower that the expected need for therapy in these services.

Note, this estimate assumes that all people living with mild-moderate dementia are suitable for psychological therapies offered in IAPT (e.g., cognitive behavioural therapy) and that undiagnosed dementia is not present in the control sample. Moreover, the observed proportion of PLWD in our sample is likely not that much lower than what would be expected in practice when also considering IAPT access targets (15-25% of prevalent cases (1, 9)) and the lower access rates of older adults generally in IAPT (6·4% in 2015 when the target is 12% (10, 11)).

D) Propensity score matching model

		Coefficient	se	95% CI	p
Gender	Female	-·33	·06	-·44, -·22	<·001
	Missing	-1·20	·59	-2·35, -·05	·04
Ethnicity	Mixed	·43	·26	-·09, ·94	·10
	Asian	·53	·14	·26, ·81	<·001
	Black	·64	·16	·33, ·96	<·001
	Other	·67	·23	·22, 1·11	·004
	Missing	·20	·10	·01, ·39	·04
IMD decile	2	-·28	·11	-·49, -·06	·01
	3	-·27	·11	-·48, -·60	·01
	4	-·51	·12	-·74, -·28	<·001
	5	-·41	·11	-·63, -·19	<·001
	6	-·40	·11	-·62, -·18	<·001
	7	-·65	·12	-·88, -·41	<·001
	8	-·61	·12	-·84, -·37	<·001
	9	-·76	·13	-1·01, -·51	<·001
	10	-·79	·13	-1·04, -·53	<·001
	Missing	-·28	·18	-·63, ·07	·12
LTC case	Yes	·61	·07	·48, ·74	<·001
	Missing	·41	·08	·24, ·57	<·001
Appointment year	2013	·10	·29	-·46, ·66	·73
	2014	·50	·27	-·03, 1·04	·07
	2015	·85	·27	·32, 1·37	·002
	2016	1·05	·27	·53, 1·57	<·001
	2017	1·05	·27	·53, 1·57	<·001
	2018	1·07	·27	·55, 1·59	<·001
	2019	1·15	·29	·59, 1·72	<·001
Psychotropic medication	Prescribed and taking	·47	·17	·14, ·80	·01
	Not prescribed	·18	·17	-·16, ·52	·29
	Missing	·74	·18	·37, 1·10	<·001
Employment status	Unemployed	·85	·07	·71, ·99	<·001
	Missing	·38	·12	·14, ·61	·002
Age at referral		·12	·002	·11, ·12	<·001
Baseline PHQ-9		·02	·01	·01, ·04	<·001
Baseline GAD-7		-·04	·01	-·05, -·03	<·001
Waiting time 1		·02	·01	·003, ·03	·02
Waiting time 2		·01	·003	·0001, ·01	·05

E) Clinical commissioning group categories

	Category	Clinical commissioning groups (CCG)
**1**	NHS England South (South West)	NHS Bristol CCG , NHS Kernow CCG, NHS North Somerset CCG, NHS Somerset CCG, NHS South Gloucestershire CCG, NHS Northern, Eastern and Western Devon CCG, NHS South Devon and Torbay CCG
**2**	NHS England South (South East)	NHS Ashford CCG, NHS Brighton and Hove CCG, NHS Canterbury and Coastal CCG, NHS Eastbourne, Hailsham and Seaford CCG, NHS Coastal West Sussex CCG, NHS Crawley CCG, NHS Dartford, Gravesham and Swanley CCG, NHS East Surrey CCG, NHS Guildford and Waverley CCG, NHS Hastings & Rother CCG, NHS Medway CCG, NHS Horsham and Mid Sussex CCG, NHS North West Surrey CCG, NHS South Kent Coast CCG, NHS Surrey Heath CCG, NHS Swale CCG, NHS Thanet CCG, NHS Surrey Downs CCG, NHS West Kent CCG, NHS High Weald Lewes Havens CCG
**3**	NHS England South (South Central)	NHS Bracknell and Ascot CCG, NHS Chiltern CCG, NHS Newbury and District CCG, NHS North & West Reading CCG, NHS Oxfordshire CCG, NHS Slough CCG, NHS South Reading CCG, NHS Aylesbury Vale CCG, NHS Windsor, Ascot and Maidenhead CCG, NHS Wokingham CCG, NHS Bath and North East Somerset CCG, NHS Gloucestershire CCG, NHS Swindon CCG, NHS Wiltshire CCG
**4**	NHS England South (Wessex)	NHS North Hampshire CCG, NHS Fareham and Gosport CCG, NHS Isle of Wight CCG, NHS Portsmouth CCG, NHS South Eastern Hampshire CCG, NHS Southampton CCG, NHS West Hampshire CCG, NHS Dorset CCG, NHS North East Hampshire and Farnham CCG
**5**	NHS England London	NHS Barking and Dagenham CCG, NHS Barnet CCG, NHS Bexley CCG, NHS Brent CCG, NHS Bromley CCG, NHS Camden CCG, NHS City and Hackney CCG, NHS Croydon CCG, NHS Ealing CCG, NHS Enfield CCG, NHS Hounslow CCG, NHS Greenwich CCG, NHS Hammersmith and Fulham CCG, NHS Haringey CCG, NHS Harrow CCG, NHS Havering CCG, NHS Hillingdon CCG, NHS Islington CCG, NHS Kingston CCG, NHS Lambeth CCG, NHS Lewisham CCG, NHS Newham CCG, NHS Redbridge CCG, NHS Richmond CCG, NHS Southwark CCG, NHS Merton CCG, NHS Sutton CCG, NHS Tower Hamlets CCG, NHS Waltham Forest CCG, NHS Wandsworth CCG, NHS West London (Kensington and Chelsea, Queen's Park and Paddington) CCG, NHS Central London (Westminster) CCG
**6**	NHS England Midlands and East (West Midlands)	NHS Birmingham South and Central CCG, NHS Coventry and Rugby CCG, NHS Dudley CCG, NHS Herefordshire CCG, NHS Warwickshire North CCG, NHS Redditch and Bromsgrove CCG, NHS Sandwell and West Birmingham CCG, NHS Solihull CCG, NHS South Warwickshire CCG, NHS South Worcestershire CCG, NHS Walsall CCG, NHS Wolverhampton CCG, NHS Wyre Forest CCGNHS Birmingham Crosscity CCG
**7**	NHS England Midlands and East (North Midlands)	NHS Erewash CCG, NHS Hardwick CCG, NHS Mansfield and Ashfield CCG, NHS Newark & Sherwood CCG, NHS North Derbyshire CCG, NHS Nottingham City CCG, NHS Nottingham North and East CCG, NHS Nottingham West CCG, NHS Rushcliffe CCG, NHS Southern Derbyshire CCG, NHS Cannock Chase CCG, NHS East Staffordshire CCG, NHS North Staffordshire CCG, NHS Shropshire CCG, NHS South East Staffordshire and Seisdon Peninsula CCG, NHS Stafford and Surrounds CCG, NHS Stoke on Trent CCG, NHS Telford and Wrekin CCG
**8**	NHS England Midlands and East (East)	NHS Cambridgeshire and Peterborough CCG, NHS Ipswich and East Suffolk CCG, NHS Great Yarmouth & Waveney CCG, NHS Mid Essex CCG, NHS North East Essex CCG, NHS North Norfolk CCG, NHS Norwich CCG, NHS South Norfolk CCG, NHS Thurrock CCG, NHS West Essex CCG, NHS West Norfolk CCG, NHS West Suffolk CCG, NHS Basildon and Brentwood CCG, NHS Castle Point and Rochford CCG, NHS Southend CCG
**9**	NHS England Midlands and East (Central Midlands)	NHS Lincolnshire East CCG, NHS Corby CCG, NHS East Leicestershire and Rutland CCG, NHS Leicester City CCG, NHS Lincolnshire West CCG, NHS Milton Keynes CCG, NHS Nene CCG, NHS South West Lincolnshire CCG, NHS West Leicestershire CCG, NHS Bedfordshire CCG, NHS East and North Hertfordshire CCG, NHS Herts Valleys CCG, NHS Luton CCG, NHS South Lincolnshire CCG
**10**	NHS England North (Lancashire and South Cumbria)	NHS Blackburn with Darwen CCG, NHS Blackpool CCG, NHS Chorley and South Ribble CCG, NHS East Lancashire CCG, NHS Greater Preston CCG, NHS Lancashire North CCG, NHS West Lancashire CCG, NHS Fylde & Wyre CCG
**11**	NHS England North (Cumbria and North East)	NHS Darlington CCG, NHS Durham Dales, Easington and Sedgefield CCG, NHS Gateshead CCG, NHS Newcastle North and East CCG, NHS Newcastle West CCG, NHS North Durham CCG, NHS Hartlepool and Stockton-on-Tees CCG, NHS Northumberland CCG, NHS South Tees CCG, NHS South Tyneside CCG, NHS Sunderland CCG, NHS Cumbria CCG, NHS North Tyneside CCG
**12**	NHS England North (Greater Manchester)	NHS Bolton CCG, NHS Bury CCG, NHS Central Manchester CCG, NHS Oldham CCG, NHS Heywood, Middleton & Rochdale CCG, NHS Salford CCG, NHS North Manchester CCG, NHS South Manchester CCG, NHS Stockport CCG, NHS Tameside and Glossop CCG, NHS Trafford CCG, NHS Wigan Borough CCG

**13**	NHS England North (Yorkshire and Humber)	NHS Airedale, Wharfedale and Craven CCG, NHS Barnsley CCG, NHS Bassetlaw CCG, NHS Bradford Districts CCG, NHS Calderdale CCG, NHS Leeds North CCG, NHS Bradford City CCG, NHS Doncaster CCG, NHS East Riding of Yorkshire CCG, NHS Greater Huddersfield CCG, NHS Leeds West CCG, NHS Hambleton, Richmondshire and Whitby CCG, NHS Harrogate and Rural District CCG, NHS Hull CCG, NHS Leeds South and East CCG, NHS North East Lincolnshire CCG, NHS North Kirklees CCG, NHS North Lincolnshire CCG, NHS Rotherham CCG, NHS Scarborough and Ryedale CCG, NHS Sheffield CCG, NHS Vale of York CCG, NHS Wakefield CCG
**14**	NHS England North (Cheshire and Merseyside)	NHS Eastern Cheshire CCG, NHS Halton CCG, NHS Knowsley CCG, NHS South Cheshire CCG, NHS South Sefton CCG, NHS Southport and Formby CCG, NHS St Helens CCG, NHS Vale Royal CCG, NHS Warrington CCG, NHS West Cheshire CCG, NHS Wirral CCG, NHS Liverpool CCG

F) Comparison of participants with complete and missing data

		Dementia group (N = 1,549)			Control group (N = 1,943,774)		
		Complete data (N = 885)	Missing data (N = 664)		Complete data (N = 1,212,484)	Missing data (N = 731,290)	
		N (%)	N (%)	p	N (%)	N (%)	p
Gender	Male	389 (43·95)	267 (40·21)	·17	396, 726 (32·72)	242,101 (33·11)	<·001
	Female	496 (56·05)	393 (59·19)		815, 758 (67·28)	482,386 (65·96)	
Ethnicity	White	796 (89·94)	435 (65·51)	·01	1,088,587 (89·78)	504,403 (68·97)	<·001
	Mixed	7 (0·79)	9 (1·36)		25,123 (2·07)	12,458 (1·70)	
	Asian	42 (4·75)	27 (4·07)		53,032 (4·37)	29,860 (4·08)	
	Black	24 (2·71)	31 (4·67)		32,441 (2·68)	15,680 (2·14)	
	Other	16 (1·81)	6 (0·90)		13,301 (1·10)	7,657 (1·05)	
IMD decile	1	102 (11·53)	129 (19·43)	<·001	121,014 (9·98)	87,648 (11·99)	<·001
	2	89 (10·06)	94 (14·16)		131,516 (10·85)	78,292 (10·71)	
	3	117 (13·22)	67 (10·09)		134,056 (11·06)	74,658 (10·21)	
	4	87 (9·83)	67 (10·09)		135,077 (11·14)	70,330 (9·62)	
	5	101 (11·41)	52 (7·83)		126,497 (10·43)	67,308 (9·20)	
	6	93 (10·51)	59 (8·89)		121,828 (10·05)	63,832 (8·73)	
	7	73 (8·25)	51 (7·68)		117,078 (9·66)	60,114 (8·22)	
	8	81 (9·15)	39 (5·87)		113,107 (9·33)	58,540 (8·01)	
	9	74 (8·36)	30 (4·52)		110,020 (9·07)	55,011 (7·52)	
	10	68 (7·68)	27 (4·07)		102,291 (8·44)	51,349 (7·02)	
LTC case	No	299 (33·79)	94 (14·60)	·98	856,692 (70·66)	230,955 (31·58)	<·001
	Yes	586 (66·21)	185 (27·86)		355,792 (29·34)	90,976 (12·44)	
Appointment year	2012	11 (1·24)	8 (1·20)	<·001	24,269 (2·00)	36,118 (4·94)	<·001
	2013	32 (3·62)	43 (6·48)		107,508 (8·87)	110,975 (15·18)	
	2014	68 (7·68)	95 (14·31)		150,087 (12·38)	140,582 (19·22)	
	2015	130 (14·69)	148 (22·29)		196,629 (16·22)	139,794 (19·12)	
	2016	170 (19·21)	145 (21·84)		226,149 (18·65)	112,028 (15·32)	
	2017	192 (21·69)	118 (17·77)		227,165 (18·74)	92,235 (12·61)	
	2018	224 (25·31)	93 (14·01)		231,212 (19·07)	82,875 (11·33)	
	2019	58 (6·55)	14 (2·11)		49,465 (4·08)	16,683 (2·28)	
Psychotropic medication	Prescribed – not taking	25 (2·82)	14 (2·11)	·03	61,822 (5·10)	30,581 (4·18)	<·001
	Prescribed and taking	587 (66·33)	239 (35·99)		622,014 (51·30)	294,794 (40·31)	
	Not prescribed	273 (30·85)	154 (23·19)		528,648 (43·60)	227,216 (31·07)	
Employment status	Employed	644 (72·77)	241 (36·30)	·35	959,793 (79·16)	463,838 (63·43)	<·001
	Unemployed	372 (42·03)	156 (23·49)		252,691 (20·84)	151,980 (20·78)	
		Mean (SD)	Mean (SD)	p	Mean (SD)	Mean (SD)	p
Age at referral		64·46 (15·76)	67·85 (16·57)	<·001	40·21 (14·76)	40·48 (14·62)	<·001
Baseline PHQ-9		16·15 (5·85)	14·78 (5·60)	<·001	15·67 (5·60)	15·81 (5·65)	<·001
Baseline GAD-7		13·46 (5·04)	12·31 (5·13)	<·001	14·29 (4·42)	14·27 (4·50)	<·001
Waiting time 1		3·07 (3·66)	3·51 (4·83)	·04	2·86 (3·65)	3·85 (5·10)	<·001
Waiting time 2		7·82 (7·46)	5·22 (6·85)	<·001	6·86 (7·01)	6·29 (7·30)	<·001
Number of sessions		6·18 (4·10)	4·67 (3·66)	<·001	6·84 (4·65)	5·95 (4·37)	<·001

Independent t-tests were used for continuous outcomes and chi-square tests were used for categorical outcomes

G) Contextualising change in therapy outcomes in PLWD with previously published studies

Study (12)	Group	Depression measure	d	95% CI	Follow up
**MODIFY**	IAPT	PHQ9	-0·83	-·90, -·75	
**Stanley (2013)**	Control	GDS	-0·23	-·94, ·47	3 months
	Peaceful Mind Program		-0·24	-1·01, ·53	
**Guetin (2009)**	Control	GDS	0·04	-·67, ·76	4 weeks
	Music therapy		-0·59	-1·32, ·15	
**Cheng (2012)**	Control	GDS	0·04	-·76, ·84	3 months
	Tai Chi		-0·60	-1·42, ·22	
**Bailey (2016)**	Control	GDS	-0·23	-·78, ·33	6 weeks
	QAR-Depression		-0·56	-1·12, -·01	
**Williams (2008)**	Control	CSDD	-0·40	-1·21, ·41	16 weeks
	Exercise training		-0·43	-1·13, ·27	
**Study**	**Group**	**Anxiety measure**	**D**	**95% CI**	**Follow up**
**MODIFY**	IAPT	GAD7	-0·80	-·87, -·72	
**Stanley (2013)**	Control	GAI	-0·26	-·97, ·44	3 months
	Peaceful Mind Program		-0·13	-·90, ·64	
**Guetin (2009)**	Control	Hamilton Scale	-0·08	-·79, ·64	4 weeks
	Music therapy		-1·42	-2·22, -·62	

H) Multilevel models

PRIMARY	Reliable improvement					Reliable recovery					Deterioration				
	N	OR	95% CI	p	ICC	N	OR	95% CI	p	ICC	N	OR	95% CI	p	ICC
PS matched (unadjusted)	2,539	·77	·65, ·91	·002	·008	2,246	·73	·62, ·86	<·001	·002	2,534	1·38	1·04, 1·83	·02	·004
PS matched (adjusted)	2,539	·81	·68, ·97	·02	·01	2,242	·78	·65, ·93	·01	·005	2,529	1·38	1·03, 1·85	·03	·002
SECONDARY	PHQ-9 Change					GAD-7 Change					WSAS total Change				
	N	b	se	P	ICC	N	b	se	p	ICC	N	B	se	p	ICC
PS matched (unadjusted)	2,544	-1·12	·27	<·001	·004	2,544	-·87	·24	<·001	·004	1,945	-1·38	·43	·001	<·001
PS matched (adjusted)	2,544	-·94	·24	<·001	·005	2,544	-·59	·20	·004	·007	1,945	-1·34	·43	·002	<·001

I) Comparison of outcomes between age of dementia diagnosis groups (65+ vs >65 years)

	Diagnosis 65+		Diagnosis <65		
**Primary outcomes**	**Total N**	**N (%)**	**Total N**	**N (%)**	**p**
**Reliable improvement**	864	554 (64·12%)	680	397 (58·38%)	·021
**Reliable recovery**	744	341 (45·83%)	631	195 (30·90%)	<·001
**Deterioration**	864	76 (8·80%)	679	77 (11·34%)	·097
**Secondary outcomes**	**Total N**	**M (SD)**	**Total N**	**M (SD)**	**p**
**PHQ-9 Change**	865	5·37 (6·24)	684	5·07 (4·54)	·375
**GAD-7 Change**	865	4·43 (5·45)	684	4·33 (6·00)	·743
**WSAS Change**	573	3·41 (8·32)	540	4·41 (10·83)	·081

Independent t-tests were used for continuous outcomes and chi-square tests were used for categorical outcomes

J) Sensitivity analyses: Primary and secondary outcomes for PLWD<65 and matched controls

**Primary outcomes**	**Reliable improvement**				**Reliable recovery**				**Reliable Deterioration**			
	N	OR	95% CI	p	N	OR	95% CI	p	N	OR	95% CI	p
**PS matched (unadjusted)**	1,216	·75	·59, ·95	·016	1,126	·73	·58, ·94	·013	1,210	1·27	·86, 1·87	·224
**PS matched (adjusted)***	1,216	·75	·58, ·96	·023	1,126	·74	·57, ·96	·023	1,210	1·31	·87, 1·99	·196
**Secondary outcomes**	**PHQ-9 Change**				**GAD-7 Change**				**WSAS Change**			
	N	B	se	p	N	B	se	p	N	B	se	p
**PS matched (unadjusted)**	1,222	-1·09	·39	·006	1,222	-·77	·35	·027	965	-1·11	·71	·117
**PS matched (adjusted)***	1,222	-1·09	·36	·003	1,222	-·69	·31	·026	965	-1·19	·71	·094

Logistic regression models were used for primary outcomes and linear regression models were used for secondary outcomes
All 611 PLWD<65 with complete data on matched variables were able to be matched
* Adjusted for all matched variables (gender, ethnicity, employment status, LTC case, psychotropic medication, IMD decile, year of first appointment, age at referral, baseline PHQ-9, baseline GAD-7, waiting times referral to assessment, waiting time assessment to treatment) and number of IAPT sessions attended

K) Sensitivity analyses: Primary and secondary outcomes for PLWD65+ and matched controls

Primary outcomes	Reliable improvement				Reliable recovery				Reliable Deterioration			
	N	OR	95% CI	p	N	OR	95% CI	p	N	OR	95% CI	p
**PS matched (unadjusted)**	1,469	·63	·50, ·79	<·001	1,236	·68	·54, ·85	·001	1,466	1·78	1·18, 2·68	·006
**PS matched (adjusted)***	1,469	·63	·50, ·80	<·001	1,228	·69	·54, ·87	·002	1,449	1·92	1·25, 2·96	·003
**Secondary outcomes**	**PHQ-9 Change**				**GAD-7 Change**				**WSAS Change**			
	N	B	se	p	N	B	se	p	N	B	se	p
**PS matched (unadjusted)**	1,472	-1·44	·34	<·001	1,472	-1·29	·29	<·001	1,057	-·91	·51	·077
**PS matched (adjusted)***	1,472	-1·52	·29	<·001	1,472	-1·01	·25	<·001	1,057	-1·04	·52	·047

Logistic regression models were used for primary outcomes and linear regression models were used for secondary outcomes
Of the 757 PLWD diagnosed 65+ with complete data on matched variables, 21 were unable to be matched
* Adjusted for all matched variables (gender, ethnicity, employment status, LTC case, psychotropic medication, IMD decile, year of first appointment, age at referral, baseline PHQ-9, baseline GAD-7, waiting times referral to assessment, waiting time assessment to treatment) and number of IAPT sessions attende

L) Sensitivity analyses: Treatment intensity

Primary outcomes	Reliable improvement				Reliable recovery				Reliable Deterioration			
	N	OR	95% CI	p	N	OR	95% CI	p	N	OR	95% CI	p
**Low intensity only (unadjusted)**	766	·62	·45, ·84	·002	638	·66	·49, ·91	·010	765	2·02	1·17, 3·49	·012
**High intensity only (unadjusted)**	998	·70	·53, ·91	·009	916	·64	·49, ·83	·001	997	1·17	·74, 1·87	·497
**Mixed intensity (unadjusted)**	285	·73	·44, 1·22	·229	260	·81	·50, 1·31	·385	284	1·62	·68, 3·87	·280
**Low intensity only (adjusted)***	766	·60	·43, ·84	·003	638	·67	·47, ·94	·022	760	2·17	1·20, 3·95	·011
**High intensity only (adjusted)***	992	·68	·51, ·91	·010	916	·63	·47, ·83	·001	977	1·31	·79, 2·19	·298
**Mixed intensity (adjusted)***	283	·74	·41, 1·36	·338	259	·86	·50, 1·48	·575	246	1·61	·57, 4·54	·369
**Secondary outcomes**	**PHQ-9 Change**				**GAD-7 Change**				**WSAS Change**			
	N	b	se	p	N	b	se	p	N	b	se	p
**Low intensity only (unadjusted)**	768	-1·28	·46	·006	768	-·96	·42	·021	679	-1·18	·72	·102
**High intensity only (unadjusted)**	1,000	-1·53	·43	<·001	1,000	-1·16	·37	·002	864	-1·72	·66	·010
**Mixed intensity (unadjusted)**	286	-1·53	·84	·070	286	-·13	·71	·852	267	-·87	1·28	·496
**Low intensity only (adjusted)***	768	-1·49	·41	<·001	768	-·88	·35	·013	679	-1·07	·71	·131
**High intensity only (adjusted)***	1,000	-1·59	·39	<·001	1,000	-1·06	·33	·001	864	-1·74	·67	·009
**Mixed intensity (adjusted)***	286	-·98	·75	·195	286	-·03	·63	·957	267	-1·58	1·24	·205

Logistic regression models were used for primary outcomes and linear regression models were used for secondary outcomes
PS matched samples were used for all analyses
* Adjusted for all matched variables (gender, ethnicity, employment status, LTC case, psychotropic medication, IMD decile, year of first appointment, age at referral, baseline PHQ-9, baseline GAD-7, waiting time referral to assessment, waiting time assessment to treatment) and number of IAPT sessions attended

References

1. National Collaborating Centre for Mental Health. The Improving Access to Psychological Therapies Manual 2021 [Available from: https://www.england.nhs.uk/wp-content/uploads/2018/06/the-iapt-manual-v5.pdf.
2. NHS Digital. Hospital Episode Statistics (HES) 2021 [Available from: https://digital.nhs.uk/data-and-information/data-tools-and-services/data-services/hospital-episode-statistics.
3. NHS Digital. Mental Health Services Data Set 2021 [Available from: https://digital.nhs.uk/data-and-information/data-collections-and-data-sets/data-sets/mental-health-services-data-set.
4. NHS Digital. Linked HES-ONS mortality data 2020 [Available from: https://digital.nhs.uk/data-and-information/data-tools-and-services/data-services/linked-hes-ons-mortality-data.
5. NHS Digital. Psychological Therapies: A guide to IAPT data and publications 2019 [Available from: https://digital.nhs.uk/binaries/content/assets/website-assets/data-and-information/data-sets/iapt/guide-to-iapt-data-and-publications.pdf.
6. Wittenberg R, Hu B, Barraza-Araiza L, Rehill A. Projections of older people with dementia and costs of dementia care in the United Kingdom, 2019–2040. London: London School of Economics. 2019.
7. Leung DK, Chan WC, Spector A, Wong GH. Prevalence of depression, anxiety, and apathy symptoms across dementia stages: A systematic review and meta-analysis. International Journal of Geriatric Psychiatry. 2021;36(9):1330-44.
8. Zenebe Y, Akele B, Necho M. Prevalence and determinants of depression among old age: a systematic review and meta-analysis. Annals of General Psychiatry. 2021;20(1):1-19.
9. Independent Mental Health Taskforce. The Five Year Forward View for Mental Health: NHS England; 2016 [Available from: https://www.england.nhs.uk/wp-content/uploads/2016/02/Mental-Health-Taskforce-FYFV-final.pdf.
10. NHS Digital. Psychological Therapies: Annual Report on the Use of IAPT Services, England 2015–16. 2016.
11. Department of Health. Talking Therapies: A Four Year Plan of Action. 2011.
12. Noone D, Stott J, Aguirre E, Llanfear K, Spector A. Meta-analysis of psychosocial interventions for people with dementia and anxiety or depression. Aging & mental health. 2019;23(10):1282-91.
